# Aerobic exercise training‐induced follistatin‐like 1 secretion in the skeletal muscle is related to arterial stiffness via arterial NO production in obese rats

**DOI:** 10.14814/phy2.15300

**Published:** 2022-05-18

**Authors:** Kenichiro Inoue, Shumpei Fujie, Naoki Horii, Henry Yamazaki, Masataka Uchida, Motoyuki Iemitsu

**Affiliations:** ^1^ Faculty of Sport and Health Science Ritsumeikan University Shiga Japan; ^2^ Research Fellow of Japan Society for the Promotion of Science Tokyo Japan; ^3^ Global Innovation Research Organization Ritsumeikan University Shiga Japan

**Keywords:** aerobic exercise training, follistatin‐like 1, nitric oxide, obesity, skeletal muscle fiber type

## Abstract

Follistatin‐like 1 (FSTL1), which is mainly secreted from skeletal muscle and myocardium, upregulates protein kinase B (Akt) and endothelial nitric oxide synthase (eNOS) phosphorylation in vascular endothelial cells. It is unclear whether skeletal muscle‐ and myocardium‐derived FSTL1 secretion induced by aerobic exercise training is involved in the reduction of arterial stiffness via arterial NO production in obese rats. This study aimed to clarify whether aerobic exercise training‐induced FSTL1 secretion in myocardium and skeletal muscle is associated with a reduction in arterial stiffness via arterial Akt‐eNOS signaling pathway in obese rats. Sixteen Otsuka Long‐Evans Tokushima Fatty (OLETF) obese rats were randomly divided into two groups: sedentary control (OLETF‐CON) and eight‐week aerobic exercise training (treadmill for 60min at 25m/min, 5days/week, OLETF‐AT). Eight Long‐Evans Tokushima Otsuka (LETO) rats were used as a healthy sedentary control group. In OLETF‐CON, serum FSTL1, arterial Akt and eNOS phosphorylation, and arterial nitrite/nitrate (NOx) levels were significantly lower, and carotid‐femoral pulse wave velocity (cfPWV) was significantly greater than those in LETO. These parameters were improved in the OLETF‐AT compared to the OLETF‐CON. In the OLETF‐AT, FSTL1 levels in slow‐twitch fiber‐rich soleus muscle were significantly greater than those in the OLETF‐CON, but not in myocardium, fast‐twitch fiber‐rich tibialis anterior muscle, and adipose tissue. Serum FSTL1 levels were positively correlated with soleus FSTL1, arterial eNOS phosphorylation, and NOx levels and negatively correlated with cfPWV. Thus, aerobic exercise training‐induced FSTL1 secretion in slow‐twitch fiber‐rich muscles may be associated with a reduction in arterial stiffness via arterial NO production in obese rats.

## Graphical abstract

In obese rats, aerobic exercise training increased FSTL1 levels in slow‐twitch fiber‐rich soleus muscle and serum but not in myocardium, fast‐twitch fiber‐rich tibialis anterior muscle, and adipose tissue. Serum FSTL1 levels were positively correlated with soleus FSTL1, arterial eNOS phosphorylation levels, and arterial NOx production and were negatively correlated with cfPWV. Our findings suggest that aerobic exercise training‐induced FSTL1 secretion in slow‐twitch fiber‐rich muscle may be associated with a reduction in arterial stiffness via arterial NO production in obese rats.

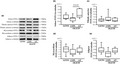

## 1 INTRODUCTION

Obesity accompanied by excessive fat accumulation causes arterial stiffness via endothelial dysfunction and vascular hypertrophy, leading to increased cardiovascular disease mortality (Aroor et al., 2018; Zong et al., 2016). However, habitual aerobic exercise reduces arterial stiffness, and its underlying mechanism is associated with vasodilation by nitric oxide (NO) production via activation of endothelial NO synthase (eNOS) phosphorylation in the arteries (Fujie et al., 2021; Hasegawa et al., 2018). Thus, aerobic exercise training‐induced arterial NO production is important for the primary prevention of cardiovascular disease in obesity. However, the regulating factor associated with aerobic exercise training‐induced increase in arterial NO production remains unclear.

Follistatin‐like 1 (FSTL1) is an endocrine hormone that belongs to the follistatin family and is mainly secreted from the skeletal muscle and the myocardium (Görgens et al., 2013; Hayakawa et al., 2015; Miyabe et al., 2014; Oshima et al., 2008). FSTL1 improves cardiac function and suppresses vascular hypertrophy, resulting in cardioprotective effects (Miyabe et al., 2014; Shimano et al., 2011). Importantly, FSTL1 transfection promotes phosphorylation of protein kinase B (Akt) and eNOS in human umbilical vein endothelial cells (HUVECs) (Ouchi et al., 2008). In addition, the binding of FSTL1 to disco interacting protein 2 homolog A (DIP2A) elevates Akt phosphorylation in HUVECs (Ouchi et al., 2010). Therefore, FSTL1 activates the arterial Akt‐eNOS signaling pathway by binding to DIP2A in vascular endothelial cells (Mattiotti et al., 2018).

A recent study showed that obesity reduces circulating FSTL1 levels (Horak et al., 2018). On the other hand, in myocardial infarction model rats, intermittent aerobic exercise training increases circulating FSTL1 levels and concomitantly increases the expression levels of skeletal muscle and myocardial FSTL1 protein (Xi et al., 2016). Thus, we hypothesized that the increase in skeletal muscle‐ and myocardium‐derived FSTL1 secretion induced by aerobic exercise training is involved in the reduction of arterial stiffness via the arterial Akt‐eNOS signaling pathway in obese rats. This study examined the effects of eight‐week aerobic exercise training on skeletal muscle‐ and myocardium‐derived FSTL1 secretion in obese rats. We investigated the relationship between circulating FSTL1 levels and arterial NO production via Akt‐eNOS or arterial stiffness. Moreover, we measured FSTL1 expression in slow‐twitch fiber‐rich soleus and fast‐twitch fiber‐rich tibialis anterior muscle because different muscle fiber types may differ from the FSTL1 secretory response to exercise training (Norheim et al., 2011).

## 2 METHODS

### 2.1 Animals and protocol

This study was approved by the Committee on Animal Care at Ritsumeikan University and conducted according to the Guiding Principles for the Care and Use of Animals, based on the Declaration of Helsinki (Helsinki, Finland). Six‐week‐old male Otsuka Long‐Evans Tokushima Fatty (OLETF) rats (Japan SLC, Shizuoka, Japan) were used as a rat model of obesity. OLETF rats were individually housed in cages under controlled conditions (12:12 h light: dark cycle) and were given *ad libitum* access to water and fed a standard laboratory diet (CE‐2; CLEA Japan, Tokyo, Japan) during the experimental period. After 14 weeks, the 20‐week‐old OLETF rats were randomly divided into the following two experimental groups: (1) Sedentary control (OLETF‐CON, *n* = 8) group and (2) the aerobic exercise training (OLETF‐AT, *n* = 8) group. In addition, age‐matched Long‐Evans Tokushima Otsuka (LETO) rats (*n* = 8, Japan SLC) were used as a healthy sedentary control group. Post‐treatment experiments and sacrifice of OLETF‐AT were performed more than 48 h after the last aerobic exercise session to avoid any acute effects of the aerobic exercise training intervention. In addition, food was removed from all cages for 12 h before measuring the bodyweight and carotid‐femoral pulse wave velocity (cfPWV; examined as an index of central arterial stiffness) of rats in all groups under general anesthesia. After that, blood samples were obtained from the abdominal aorta. After sacrifice, the epididymal fat, soleus muscle, tibialis anterior muscle, left ventricle, and aorta were quickly resected, rinsed in ice‐cold saline, weighed, and frozen in liquid nitrogen. All samples were stored at −80°C for further analysis.

### 2.2 Aerobic exercise training protocol

We used the same aerobic exercise training program, which consisted of moderate‐intensity treadmill running, according to the methods of previous studies with minor modifications (Horii et al., 2019; Sato et al., 2017; Wang et al., 2020). Before the training experimental period, the OLETF‐AT group exercised on a treadmill at 10–15 m/min for three days to get used to the exercise. The OLETF‐AT group ran on the treadmill for 1 h at 25 m/min, without incline, 5 days/week for eight weeks beginning at 20 weeks of age. Exercise intensity remained constant during the training period.

### 2.3 Blood pressures and cfPWV

Systolic (SBP) and diastolic (DBP) blood pressure, as well as cfPWV, were measured as previously described (Hasegawa et al., 2018). Prior to sacrifice, rats were anesthetized with an intraperitoneal injection of pentobarbital sodium (0.1 ml/kg) and inhalation of 2% sevoflurane. Blood pressure and cfPWV were measured using two catheters, and an animal heating board was used to maintain the body temperature of the rats at 37°C. Catheters with pressure transducer (DT4812; Nihon Kohden, Tokyo, Japan) was implanted at two points in the aortic arch via the left carotid artery (SP45, Natume, Tokyo, Japan) and in the proximal abdominal aortic bifurcation via the left femoral artery (SP31, Natume, Tokyo, Japan). Pulse pressure waves recorded using the two pressure transducers were simultaneously imported to an amplifier and displayed on a data acquisition system (PEG‐1000, Nihon Koden, Tokyo, Japan) at a sampling rate of 10,000 Hz. After that, the straight‐line distance between the tips of the two catheters was measured. The propagation time from the aortic arch to the abdominal aortic bifurcation is the difference in time between the commencement of the upstroke of each pulse waveform. The cfPWV was calculated by dividing the propagation distance by the propagation time, and SBP and DBP were simultaneously measured using pulse pressure waves in the left carotid artery.

### 2.4 Fasting glucose and insulin concentrations

Fasting glucose was assessed in the tail vein after overnight fasting. Glucose concentrations were assessed three times using a blood glucose meter (Terumo Corporation, Tokyo, Japan). Serum insulin concentrations were measured using an enzyme‐linked immunosorbent assay kit (Shibayagi, Gunma, Japan). Optical density at 450 nm was quantified using a microplate reader (Horii et al., 2019).

### 2.5 Insulin sensitivity

As an index of insulin sensitivity, the quantitative insulin sensitivity check index (QUICKI) was calculated from fasting glucose and insulin concentrations based on a previous study (Horii et al., 2019).

### 2.6 Citrate synthase (CS) activity

CS activity, a marker of aerobic exercise training adaptation (Vigelsø et al., 2014), was investigated using slow‐twitch fiber‐rich soleus muscles recruited by aerobic exercise training. Soleus muscle tissues of the three groups were homogenized in ten volumes of 250 mM sucrose, 1 mM Tris‐HCl (pH 7.4), and 130 mM NaCl on ice using a Teflon homogenizer. After that, CS activity was measured at 412 nm for 3 min using a spectrophotometer (xMark microplate spectrophotometer, Bio‐Rad Laboratories, Hercules, CA, USA) as previously described (Horii et al., 2019; Sato et al., 2011).

### 2.7 Immunoblot analysis

Western blot analysis was performed as described previously (Hasegawa et al., 2018; Horii et al., 2019). Briefly, arterial (20 µg protein), soleus, tibialis anterior, left ventricle (40 µg protein), adipose (10 µg protein) proteins, and serum (1 µL) were separated by 10% SDS‐PAGE and then transferred to polyvinylidene difluoride membranes (Millipore, Billerica, MA, USA). The membranes were treated with blocking buffer (3% serum FSTL1, 2% soleus, tibialis anterior, and left ventricle FSTL1, DIP2A, phospho‐Akt, total Akt, phospho‐eNOS, and total eNOS, or 1% adipose FSTL1 in skim milk in PBS with 0.1% Tween 20 [PBS‐T]) for 1 h at room temperature. The membranes were incubated with antibodies against FSTL1 (1:1000; Cat. No. ab11805, Abcam, Cambridge, UK), DIP2A (1:1000; Cat. No. LS‐C356726, LifeSpan Biosciences, Seattle, WA, USA), phospho‐Akt (Ser473, 1:1000; Cat. No. 9271S, Cell Signaling Technology, Danvers, MA, USA), total Akt (1:1000; Cat. No. 9272S, Cell Signaling Technology), phospho‐eNOS (Ser1177, 1:500; Cat. No. sc‐12972‐R, Santa Cruz Biotechnology, Dallas, TX, USA), and total eNOS (1:500; Cat. No. 610297, BD Biosciences, Franklin Lakes, NJ, USA) diluted in blocking buffer for 12 h at 4°C. α‐Tubulin protein (1: 1000; Cat. No. PM054, MBL, Tokyo, Japan) was used as the loading control. After washing three times with PBS‐T, the membranes were incubated with horseradish peroxidase‐conjugated anti‐goat (FSTL1; 1:3000; Cat. No. 611620, Invitrogen, Waltham, MA, USA), anti‐rabbit (DIP2A and α‐tubulin; 1:3000; Cat. No. NA9340V, GE Healthcare UK Ltd, Buckinghamshire, UK; phospho‐Akt and total Akt; 1:3000; Cat. No. 7074S, Cell Signaling Technology) or anti‐mouse (phospho‐eNOS and total eNOS; 1:3000; Cat. No. 7076S, GE Healthcare UK Ltd) immunoglobulin diluted in blocking buffer for 1 h at room temperature. After that, the membranes were washed with PBS‐T three times. Finally, protein levels were detected using the Enhanced Chemiluminescence Plus System (GE Healthcare UK Ltd) and visualized on a LAS4000 imager (GE Healthcare Bio‐Sciences AB, Uppsala, Sweden) or FUSION FX7 EDGE (Vilber Lourmat, Collégien, France).

### 2.8 Griess assay

The arterial nitrite/nitrate (NOx) concentrations were measured using the Griess assay (R&D Systems, Minneapolis, MN, USA). The optical density was measured at 540 nm using a microplate reader (xMark microplate spectrophotometer, Bio‐Rad Laboratories). The values were converted into concentrations using a linear fit of the log‐log plot of the standard curve (Hasegawa et al., 2018).

### 2.9 Statistical analysis

Values are expressed as the mean ± standard deviation (SD). A one‐way analysis of variance (ANOVA) was used to compare differences between the LETO, OLETF‐CON, and OLETF‐AT groups. A post hoc comparison test was used to correct for multiple comparisons (Fisher's test) when ANOVA analyses showed significant differences. The relationships between the serum FSTL1 levels and the soleus, tibialis anterior, myocardium, adipose FSTL1 protein levels, eNOS phosphorylation levels, arterial NOx levels, or cfPWV, and arterial NOx levels and cfPWV in the three groups were determined using Pearson correlation coefficients. A *p* < 0.05 was considered statistically significant. All statistical analyses were performed using Stat View (5.0; SAS Institute, Tokyo, Japan).

## 3 RESULTS

### 3.1 Animal characteristics

**FIGURE 1:**
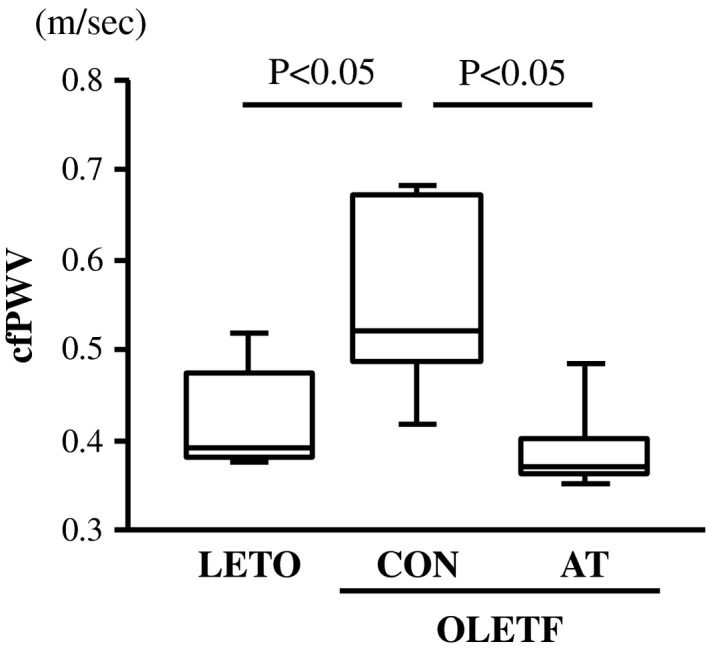
Carotid‐femoral pulse wave velocity (cfPWV) in the LETO, OLETF‐CON, and OLETF‐AT groups. One‐way ANOVA followed by Fisher's post hoc test. *n* = 5 in each group

Bodyweight, epididymal fat mass, fasting blood glucose levels, and fasting insulin levels were significantly greater (*p* < 0.05, Table 1), and the left ventricular mass, soleus muscle mass, tibialis anterior muscle mass, soleus CS activity, and QUICKI were significantly lower in the OLETF‐CON group compared to the LETO group (*p* < 0.05, Table 1). Compared with the OLETF‐CON group, bodyweight, epididymal fat mass, fasting blood glucose levels, and fasting insulin levels were significantly lower (*p* < 0.05, Table 1), and the left ventricular mass, soleus muscle mass, tibialis anterior muscle mass, soleus CS activity, and QUICKI were significantly greater in the OLETF‐AT group (*p* < 0.05, Table 1). No significant differences in heart rate, SBP, and DBP were observed among the three groups (Table 1). Furthermore, cfPWV was significantly greater in the OLETF‐CON group than in the LETO group (*p* < 0.05, Figure 1) and lower in the OLETF‐AT group than in the OLETF‐CON group (*p* < 0.05, Figure 1).

**TABLE 1:** Animal characteristic

	LETO	OLETF‐CON	OLETF‐AT
Body weight (g)	498.4 ± 24.6	609.6 ± 22.9*	477.9 ± 29.1**
Epididymal fat mass/BW (mg/g, BW)	14.7 ± 2.0	19.6 ± 4.4*	12.5 ± 4.3**
Left ventricular mass/BW (mg/g, BW)	2.46 ± 0.19	2.16 ± 0.30*	2.56 ± 0.35**
Tibialis anterior muscle mass/BW (mg/g, BW)	1.39 ± 0.15	1.15 ± 0.14*	1.33 ± 0.23**
Soleus muscle mass/BW (mg/g, BW)	0.45 ± 0.02	0.34 ± 0.02*	0.45 ± 0.05**
Soleus CS activity (µmol/g/min)	18.0 ± 4.9	12.5 ± 2.6*	24.0 ± 3.4* ^,^ **
Heart rate (beats/min)	339.5 ± 41.4	354.7 ± 24.5	339.8 ± 21.1
SBP (mmHg)	98.0 ± 13.1	106.0 ± 11.5	98.7 ± 4.8
DBP (mmHg)	71.0 ± 11.0	78.7 ± 6.1	76.3 ± 5.4
Fasting blood glucose levels (mmol/l)	6.0 ± 0.9	20.0 ± 1.5*	8.0 ± 1.2* ^,^ **
Fasting insulin levels (pmol/l)	1.28 ± 0.15	11.0 ± 2.8*	7.3 ± 0.6* ^,^ **
QUICKI (A.U.)	0.45 ± 0.02	0.27 ± 0.01*	0.32 ± 0.01* ^,^ **

Note: Values are mean ± standard deviation (SD).

Abbreviations: BW, body weight; CS, citrate synthase; DBP, diastolic blood pressure; LETO, healthy‐sedentary control group; OLETF‐CON, OLETF‐sedentary control group; OLETF‐AT, OLETF‐aerobic exercise training group; QUICKI, quantitative insulin sensitivity check index; SBP, systolic blood pressure.

* *p* < 0.05 vs. LETO

** *p* < 0.05 vs. OLETF‐CON.

### 3.2 FSTL1 levels in the serum, skeletal muscle, myocardium, and adipose tissue

**FIGURE 2:**
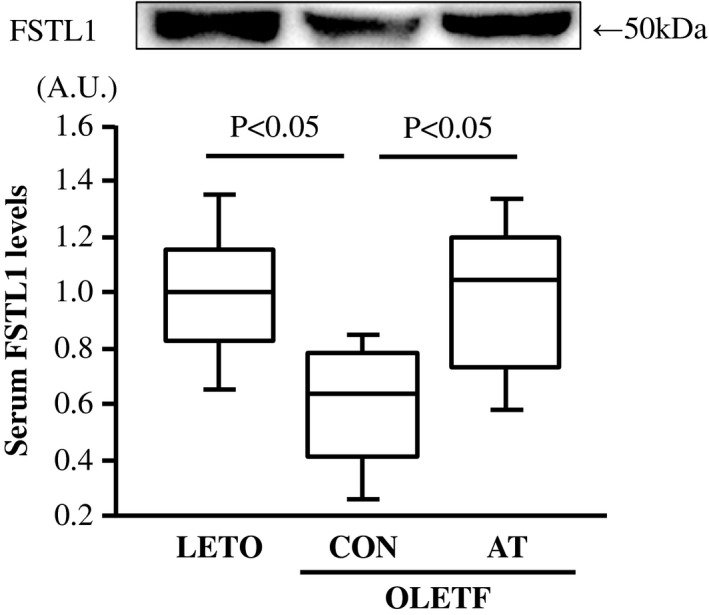
Serum follistatin‐like 1 (FSTL1) levels in the LETO, OLETF‐CON, and OLETF‐AT groups. Representative immunofluorescence image of serum FSTL1 is shown. The relative serum FSTL1 levels are represented as fold changes from the LETO group. A.U.: Arbitrary units. One‐way ANOVA followed by Fisher's post hoc test. *n* = 8 in each group

**FIGURE 3:**
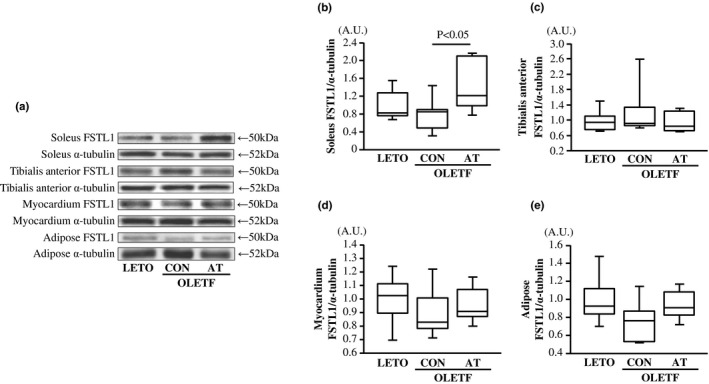
Soleus (a–b), tibialis anterior (a, c), myocardium (a, d), and adipose (a, e) follistatin‐like 1 (FSTL1) levels in the LETO, OLETF‐CON, and OLETF‐AT groups. Representative immunofluorescence images of soleus, tibialis anterior, myocardium, adipose FSTL1, and α‐tubulin are shown (a). The relative soleus, tibialis anterior, myocardium, and adipose FSTL1 levels are represented as fold changes from the LETO group. A.U.: Arbitrary units. One‐way ANOVA followed by Fisher's post hoc test. *n* = 8 in each group

Serum FSTL1 levels were significantly lower in the OLETF‐CON group than in the LETO group (*p* < 0.05, Figure 2). Serum and soleus FSTL1 levels were significantly higher in the OLETF‐AT group than in the OLETF‐CON group (*p* < 0.05, Figures 2, 3a–b). There were no significant differences in the tibialis anterior, myocardium, and adipose FSTL1 levels among the three groups (Figure 3a,c–e).

### 3.3 Arterial DIP2A, Akt, eNOS phosphorylation, and arterial NOx levels

**FIGURE 4:**
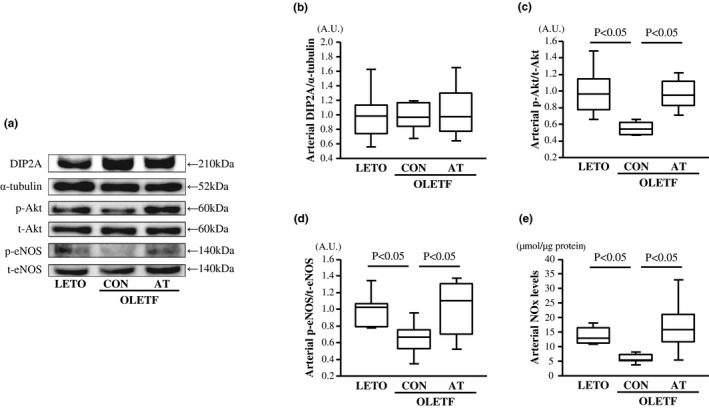
Arterial disco interacting protein 2 homolog A (DIP2A) (a–b) levels, arterial protein kinase B (Akt) (a, c), endothelial nitric oxide synthase (eNOS) (a, d) phosphorylation levels, and arterial nitrite/nitrate (NOx) levels (e) in the LETO, OLETF‐CON, and OLETF‐AT groups. Representative immunofluorescence images of DIP2A, α‐tubulin, p‐Akt, t‐Akt, p‐eNOS, and t‐eNOS are shown (a). The relative arterial DIP2A level, arterial Akt and eNOS phosphorylation levels are represented as fold changes from the LETO group. A.U.: Arbitrary units. One‐way ANOVA followed by Fisher's post hoc test. *n* = 8 in each group

No significant differences in arterial DIP2A levels were observed among the three groups (Figure 4a–b). Arterial Akt and eNOS phosphorylation levels and arterial NOx levels were significantly lower in the OLETF‐CON group than in the LETO group (*p* < 0.05, Figure 4a,c–e), and higher in the OLETF‐AT group than in the OLETF‐CON group (*p* < 0.05, Figure 4a,c–e).

### 3.4 Relationship between serum FSTL1 levels and other variables

**FIGURE 5:**
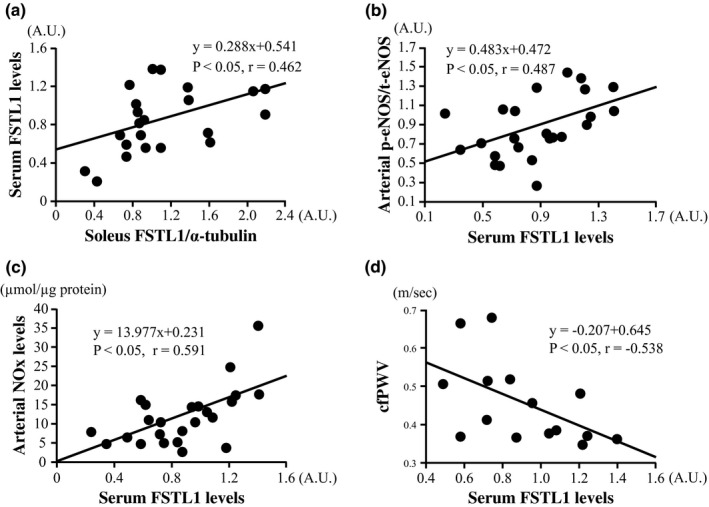
Correlations between serum FSTL1 and soleus FSTL1 levels (a), arterial endothelial nitric oxide synthase (eNOS) phosphorylation levels (b), arterial nitrite/nitrate (NOx) levels (c), or carotid‐femoral pulse wave velocity (cfPWV) (d) in the LETO, OLETF‐CON, and OLETF‐AT groups. *n* = 5–8 in each group

Serum FSTL1 levels were positively correlated with soleus FSTL1 levels (*p* < 0.05, *r* = 0.462, Figure 5a), arterial eNOS phosphorylation (*p* < 0.05, *r* = 0.487, Figure 5b), and NOx levels (*p* < 0.05, *r* = 0.591, Figure 5c) and were negatively correlated with cfPWV (*p* < 0.05, *r* = −0.538, Figure 5d) in the LETO, OLETF‐CON, and OLETF‐AT groups. No significant correlation between the serum FSTL1 levels and tibialis anterior, myocardium, and adipose FSTL1 levels was observed in the three groups. Arterial NOx levels were negatively correlated with cfPWV in the three groups (*p* < 0.05, *r* = −0.515).

## 4 DISCUSSION

This study found that aerobic exercise training increased serum FSTL1 levels, arterial Akt and eNOS phosphorylation levels, arterial NOx levels, and decreased cfPWV. Serum FSTL1 levels were positively correlated with arterial eNOS phosphorylation and NOx levels and negatively correlated with cfPWV. Moreover, soleus FSTL1 levels were increased by aerobic exercise training and positively correlated with serum FSTL1 levels. However, FSTL1 levels in the tibialis anterior muscle, myocardium, and adipose tissue were not changed by aerobic exercise training and were not correlated with serum FSTL1 levels. Thus, these data suggest that aerobic exercise training‐induced FSTL1 secretion in slow‐twitch muscles, such as the soleus muscle, is associated with arterial NO production via the Akt‐eNOS signaling pathway in obese rats, leading to a reduction in cfPWV.

A previous study showed that FSTL1 transfection enhances eNOS phosphorylation via Akt phosphorylation in HUVECs (Ouchi et al., 2008). FSTL1 recombinant binds to DIP2A, leading to accelerated Akt phosphorylation in HUVECs (Ouchi et al., 2010). Therefore, binding FSTL1 to DIP2A stimulates the Akt‐eNOS signaling pathway in vascular endothelial cells (Mattiotti et al., 2018). We revealed that serum FSTL1 levels, arterial Akt and eNOS phosphorylation levels, and arterial NOx levels were decreased in obese rats. In the exercised rats, serum FSTL1 levels, arterial Akt and eNOS phosphorylation levels, and arterial NOx levels were increased. Serum FSTL1 levels positively correlated with arterial eNOS phosphorylation and arterial NOx levels. Arterial DIP2A levels did not differ among the three groups. Thus, our findings suggest that obesity‐induced decrease or aerobic exercise training‐induced increase in circulating FSTL1 levels may be associated with changes in arterial NO production via the Akt‐eNOS signaling pathway without changes in the arterial FSTL1 receptor expression levels.

Aerobic exercise mainly recruits slow‐twitch fibers, largely depends on aerobic energy production, and promotes secretion of muscle‐derived factors, such as interleukin‐6, in slow‐twitch fiber‐rich soleus muscle, but not in fast‐twitch fiber‐rich extensor digitorum longus muscle (D'Amico et al., 2021; Laursen, 2010). Furthermore, aerobic exercise training has been reported to increase lipid peroxidation, which is an oxidative damage marker in the slow‐twitch fiber‐rich soleus muscle, but not in the fast‐twitch fiber‐rich tibialis anterior muscle (Abruzzo et al., 2013). These responses to aerobic exercise are caused only in slow‐twitch muscle fibers. We examined FSTL1 expression by using two opposite types of skeletal muscle differing in fiber type composition: the soleus muscle as a slow‐twitch muscle and the tibialis anterior muscle as a fast‐twitch muscle. Slow‐twitch fiber‐rich soleus FSTL1 levels were increased by aerobic exercise training and positively correlated with serum FSTL1 levels. In contrast, fast‐twitch fiber‐rich tibialis anterior FSTL1 levels did not change and were not correlated with serum FSTL1 levels. These results suggest that the response of FSTL1 expression to aerobic exercise training was different between slow and fast‐twitch fibers. Therefore, aerobic exercise training‐induced FSTL1 expression in slow‐twitch fibers may contribute to an increase in circulating FSTL1 levels. A previous study that used muscle‐specific FSTL1 deficient and muscle‐specific FSTL1 overexpression mice confirmed that skeletal muscle is one of the main secretory sources of FSTL1 (Miyabe et al., 2014).

Cardiac myocyte‐specific FSTL1 knockout decreases circulating FSTL1 levels in a transverse aortic constriction‐induced cardiac hypertrophy mouse model (Shimano et al., 2011). A previous study reported that heart tissue is one of the secretory sources of FSTL1 (Hayakawa et al., 2015; Oshima et al., 2008). Interval aerobic exercise training (treadmill running, alternated between 7 min at 25 m/min and 3 min at 15 m/min)‐induced increase in myocardial FSTL1 expression attenuates cardiac dysfunction in a rat model of myocardial infarction (Xi et al., 2016). However, in this study, 25 m/min treadmill running training did not affect myocardial FSTL1 levels. In the obese rats in this study, severe cardiac dysfunction was probably not caused because the left ventricle mass did not increase compared with healthy rats. Since myocardial infarction decreases maximal oxygen uptake (Rodrigues et al., 2013), the relative intensity of aerobic exercise in this study may be lower than that in a previous study. Therefore, the intensity of exercise in this study may be insufficient to increase myocardial FSTL1 expression in obese rats.

Obesity has been reported to decrease serum FSTL1 levels in both the present and previous studies (Horak et al., 2018). However, no significant differences in the myocardium, soleus, tibialis anterior, and adipose FSTL1 levels between obese and non‐obese rats were found. The obesity‐induced decrease in circulating FSTL1 levels is not explained by the skeletal muscle, myocardium or adipose FSTL1 expression. FSTL1 is expressed in the myocardium, skeletal muscle, adipose tissue, and multiple organs (Adams et al., 2010). Therefore, low FSTL1 expression in other organs may be associated with a reduction in circulating FSTL1 levels in obesity.

Lower circulating FSTL1 levels in patients with severe obesity than in non‐obese adults and lower adipose FSTL1 expression levels in obese mice than in healthy mice have been observed (Horak et al., 2018; Prieto‐Echagüe et al., 2017). Conversely, serum FSTL1 levels in overweight/obese patients compared to healthy adults and adipose FSTL1 expression levels in db/db mice compared with wild‐type mice were increased (Xu et al., 2020). In the present study, adipose FSTL1 expression levels did not change among healthy‐sedentary, obese‐sedentary, and obese‐exercise training rats, but circulating FSTL1 levels decreased in obese‐sedentary OLETF rats. Therefore, because of the discrepancy in FSTL1 expression responses to obesity, the difference in genetic background or severity of obesity may be associated with transcriptional and translational regulation of FSTL1 levels.

In this study, aerobic exercise training‐induced increases in serum FSTL1 levels were positively correlated with arterial eNOS phosphorylation and NOx levels. Various hormonal factors are involved in the regulation of NO production (Duckles & Miller, 2010; Sudar‐Milovanovic et al., 2017). To clarify whether FSTL1 contributes to low arterial stiffness via increased NO production, the effect of exercise training on arterial stiffness should be examined using skeletal muscle‐specific FSTL1 knockout mice. In this study, we showed that aerobic exercise‐induced FSTL1 secretion might be involved in increased NO production via activation of the arterial Akt‐eNOS pathway. The Akt‐eNOS pathway is also regulated by irisin, an exercise‐induced myokine (Inoue et al., 2020). However, as FSTL1 may be involved in increasing NO production via GDF‐15, FSTL1 may affect NO production via multiple pathways (Eddy & Trask, 2021; Ha et al., 2019; Widera et al., 2012). Therefore, further studies are needed to elucidate the regulation of NO production by FSTL1. KLF‐15 and c‐Jun are transcription regulating factors of FSTL1 (Wu et al., 2010; Zheng et al., 2017). Therefore, further studies are needed to examine these transcription factors in response to exercise training.

In conclusion, we demonstrated that aerobic exercise training increases serum FSTL1 levels with an elevation of FSTL1 expression in the soleus muscle but not in the myocardium, tibialis anterior muscle, and adipose tissue. Thus, aerobic exercise training may promote FSTL1 secretion in slow‐twitch fiber‐rich muscles. We also confirmed a significant association between circulating FSTL1 levels and arterial NO production and cfPWV. Therefore, aerobic exercise training‐induced increase in FSTL1 secretion is related to the reduction of arterial stiffness via arterial NO production in obese rats.

## CONFLICTS OF INTEREST

The authors declare no conflicts of interest.

## ETHICS STATEMENT

This study was approved by the Committee on Animal Care at Ritsumeikan University and conducted according to the Guiding Principles for the Care and Use of Animals, based on the Declaration of Helsinki (Helsinki, Finland).

## AUTHOR CONTRIBUTIONS

Motoyuki Iemitsu and Kenichiro Inoue conceived and designed the research; Motoyuki Iemitsu, Kenichiro Inoue, Shumpei Fujie, Naoki Horii, Henry Yamazaki, and Masataka Uchida performed the experiments; Kenichiro Inoue and Shumpei Fujie analyzed the data; Motoyuki Iemitsu, Kenichiro Inoue, Shumpei Fujie, Naoki Horii, Henry Yamazaki, and Masataka Uchida interpreted the results of the experiments; Kenichiro Inoue and Shumpei Fujie prepared the figures; Motoyuki Iemitsu, Kenichiro Inoue, Shumpei Fujie, Naoki Horii, Henry Yamazaki, and Masataka Uchida drafted the manuscript; Motoyuki Iemitsu, Kenichiro Inoue, Shumpei Fujie, Naoki Horii, Henry Yamazaki, and Masataka Uchida approved the final version of the manuscript.

## References

[phy215300-bib-0001] Abruzzo, P. M. , Esposito, F. , Marchionni, C. , Di Tullio, S. , Belia, S. , Fulle, S. , Veicsteinas, A. , & Marini, M. (2013). Moderate exercise training induces ROS‐related adaptations to skeletal muscles. International Journal of Sports Medicine, 34, 676–687. 10.1055/s-0032-1323782 23325712

[phy215300-bib-0002] Adams, D. C. , Karolak, M. J. , Larman, B. W. , Liaw, L. , Nolin, J. D. , & Oxburgh, L. (2010). Follistatin‐like 1 regulates renal IL‐1beta expression in cisplatin nephrotoxicity. American Journal of Physiology‐Renal Physiology, 299, F1320–F1327. 10.1152/ajprenal.00325.2010 20861081 PMC3006315

[phy215300-bib-0003] Aroor, A. R. , Jia, G. , & Sowers, J. R. (2018). Cellular mechanisms underlying obesity‐induced arterial stiffness. American Journal of Physiology‐Regulatory, Integrative and Comparative Physiology, 314, R387–R398. 10.1152/ajpregu.00235.2016 29167167 PMC5899249

[phy215300-bib-0004] D'amico, D. , Marino Gammazza, A. , Macaluso, F. , Paladino, L. , Scalia, F. , Spinoso, G. , Dimauro, I. , Caporossi, D. , Cappello, F. , Di Felice, V. , & Barone, R. (2021). Sex‐based differences after a single bout of exercise on PGC1alpha isoforms in skeletal muscle: A pilot study. FASEB Journal, 35, e21328. 10.1096/fj.202002173R 33433932

[phy215300-bib-0005] Duckles, S. P. , & Miller, V. M. (2010). Hormonal modulation of endothelial NO production. Pflugers Archiv: European Journal of Physiology, 459, 841–851. 10.1007/s00424-010-0797-1 20213497 PMC2865573

[phy215300-bib-0006] Eddy, A. C. , & Trask, A. J. (2021). Growth differentiation factor‐15 and its role in diabetes and cardiovascular disease. Cytokine & Growth Factor Reviews, 57, 11–18. 10.1016/j.cytogfr.2020.11.002 33317942 PMC7897243

[phy215300-bib-0007] Fujie, S. , Hasegawa, N. , Horii, N. , Inoue, K. , Uchida, M. , & Iemitsu, M. (2021). Effects of combined exercise training and Chlorella intake on vasorelaxation mediated by nitric oxide in aged mice. Applied Physiology, Nutrition, and Metabolism, 46, 479–484. 10.1139/apnm-2020-0543 33186051

[phy215300-bib-0008] Görgens, S. W. , Raschke, S. , Holven, K. B. , Jensen, J. , Eckardt, K. , & Eckel, J. (2013). Regulation of follistatin‐like protein 1 expression and secretion in primary human skeletal muscle cells. Archives of Physiology and Biochemistry, 119, 75–80. 10.3109/13813455.2013.768270 23419164

[phy215300-bib-0009] Ha, G. , De Torres, F. , Arouche, N. , Benzoubir, N. , Ferratge, S. , Hatem, E. , Anginot, A. , & Uzan, G. (2019). GDF15 secreted by senescent endothelial cells improves vascular progenitor cell functions. PLoS One, 14, e0216602. 10.1371/journal.pone.0216602 31075112 PMC6510423

[phy215300-bib-0010] Hasegawa, N. , Fujie, S. , Horii, N. , Miyamoto‐Mikami, E. , Tsuji, K. , Uchida, M. , Hamaoka, T. , Tabata, I. , & Iemitsu, M. (2018). Effects of different exercise modes on arterial stiffness and nitric oxide synthesis. Medicine & Science in Sports & Exercise, 50, 1177–1185. 10.1249/MSS.0000000000001567 29381650

[phy215300-bib-0011] Hayakawa, S. , Ohashi, K. , Shibata, R. , Kataoka, Y. , Miyabe, M. , Enomoto, T. , Joki, Y. , Shimizu, Y. , Kambara, T. , Uemura, Y. , Yuasa, D. , Ogawa, H. , Matsuo, K. , Hiramatsu‐Ito, M. , Van Den Hoff, M. J. , Walsh, K. , Murohara, T. , & Ouchi, N. (2015). Cardiac myocyte‐derived follistatin‐like 1 prevents renal injury in a subtotal nephrectomy model. Journal of the American Society of Nephrology, 26, 636–646. 10.1681/ASN.2014020210 25071081 PMC4341480

[phy215300-bib-0012] Horak, M. , Kuruczova, D. , Zlamal, F. , Tomandl, J. , & Bienertova‐Vasku, J. (2018). Follistatin‐like 1 is downregulated in morbidly and super obese central‐european population. Disease Markers, 2018, 4140815. 10.1155/2018/4140815 30595761 PMC6282119

[phy215300-bib-0013] Horii, N. , Hasegawa, N. , Fujie, S. , Uchida, M. , Iemitsu, K. , Inoue, K. , & Iemitsu, M. (2019). Effect of combination of chlorella intake and aerobic exercise training on glycemic control in type 2 diabetic rats. Nutrition, 63–64, 45–50. 10.1016/j.nut.2019.01.008 30928787

[phy215300-bib-0014] Inoue, K. , Fujie, S. , Hasegawa, N. , Horii, N. , Uchida, M. , Iemitsu, K. , Sanada, K. , Hamaoka, T. , & Iemitsu, M. (2020). Aerobic exercise training‐induced irisin secretion is associated with the reduction of arterial stiffness via nitric oxide production in adults with obesity. Applied Physiology, Nutrition, and Metabolism, 45, 715–722. 10.1139/apnm-2019-0602 31860334

[phy215300-bib-0015] Laursen, P. B. (2010). Training for intense exercise performance: High‐intensity or high‐volume training? Scandinavian Journal of Medicine & Science in Sports, 20(Suppl 2), 1–10. 10.1111/j.1600-0838.2010.01184.x 20840557

[phy215300-bib-0016] Mattiotti, A. , Prakash, S. , Barnett, P. , & Van Den Hoff, M. J. B. (2018). Follistatin‐like 1 in development and human diseases. Cellular and Molecular Life Sciences, 75, 2339–2354. 10.1007/s00018-018-2805-0 29594389 PMC5986856

[phy215300-bib-0017] Miyabe, M. , Ohashi, K. , Shibata, R. , Uemura, Y. , Ogura, Y. , Yuasa, D. , Kambara, T. , Kataoka, Y. , Yamamoto, T. , Matsuo, K. , Joki, Y. , Enomoto, T. , Hayakawa, S. , Hiramatsu‐Ito, M. , Ito, M. , Van Den Hoff, M. J. , Walsh, K. , Murohara, T. , & Ouchi, N. (2014). Muscle‐derived follistatin‐like 1 functions to reduce neointimal formation after vascular injury. Cardiovascular Research, 103, 111–120. 10.1093/cvr/cvu105 24743592 PMC4834864

[phy215300-bib-0018] Norheim, F. , Raastad, T. , Thiede, B. , Rustan, A. C. , Drevon, C. A. , & Haugen, F. (2011). Proteomic identification of secreted proteins from human skeletal muscle cells and expression in response to strength training. American Journal of Physiology‐Endocrinology and Metabolism, 301, E1013–E1021. 10.1152/ajpendo.00326.2011 21828336

[phy215300-bib-0019] Oshima, Y. , Ouchi, N. , Sato, K. , Izumiya, Y. , Pimentel, D. R. , & Walsh, K. (2008). Follistatin‐like 1 is an Akt‐regulated cardioprotective factor that is secreted by the heart. Circulation, 117, 3099–3108. 10.1161/CIRCULATIONAHA.108.767673 18519848 PMC2679251

[phy215300-bib-0020] Ouchi, N. , Asaumi, Y. , Ohashi, K. , Higuchi, A. , Sono‐Romanelli, S. , Oshima, Y. , & Walsh, K. (2010). DIP2A functions as a FSTL1 receptor. Journal of Biological Chemistry, 285, 7127–7134. 10.1074/jbc.M109.069468 20054002 PMC2844162

[phy215300-bib-0021] Ouchi, N. , Oshima, Y. , Ohashi, K. , Higuchi, A. , Ikegami, C. , Izumiya, Y. , & Walsh, K. (2008). Follistatin‐like 1, a secreted muscle protein, promotes endothelial cell function and revascularization in ischemic tissue through a nitric‐oxide synthase‐dependent mechanism. Journal of Biological Chemistry, 283, 32802–32811. 10.1074/jbc.M803440200 18718903 PMC2583310

[phy215300-bib-0022] Prieto‐Echagüe, V. , Lodh, S. , Colman, L. , Bobba, N. , Santos, L. , Katsanis, N. , Escande, C. , Zaghloul, N. A. , & Badano, J. L. (2017). BBS4 regulates the expression and secretion of FSTL1, a protein that participates in ciliogenesis and the differentiation of 3T3‐L1. Scientific Reports, 7, 9765. 10.1038/s41598-017-10330-0 28852127 PMC5575278

[phy215300-bib-0023] Rodrigues, B. , Mostarda, C. T. , Jorge, L. , Barboza, C. A. , Grans, C. F. , De Angelis, K. , & Irigoyen, M. C. (2013). Impact of myocardial infarction on cardiac autonomic function in diabetic rats. Journal of Diabetes and Its Complications, 27, 16–22. 10.1016/j.jdiacomp.2012.08.002 23044051

[phy215300-bib-0024] Sato, K. , Fujita, S. , & Iemitsu, M. (2017). Dioscorea esculenta‐induced increase in muscle sex steroid hormones is associated with enhanced insulin sensitivity in a type 2 diabetes rat model. FASEB Journal, 31, 793–801. 10.1096/fj.201600874R 27871063

[phy215300-bib-0025] Sato, K. , Iemitsu, M. , Aizawa, K. , Mesaki, N. , & Fujita, S. (2011). Increased muscular dehydroepiandrosterone levels are associated with improved hyperglycemia in obese rats. American Journal of Physiology‐Endocrinology and Metabolism, 301, E274–E280. 10.1152/ajpendo.00564.2010 21285401

[phy215300-bib-0026] Shimano, M. , Ouchi, N. , Nakamura, K. , Van Wijk, B. , Ohashi, K. , Asaumi, Y. , Higuchi, A. , Pimentel, D. R. , Sam, F. , Murohara, T. , Van Den Hoff, M. J. , & Walsh, K. (2011). Cardiac myocyte follistatin‐like 1 functions to attenuate hypertrophy following pressure overload. Proceedings of the National Academy of Sciences of the United States of America, 108, E899–E906. 10.1073/pnas.1108559108 21987816 PMC3203781

[phy215300-bib-0027] Sudar‐Milovanovic, E. , Zafirovic, S. , Jovanovic, A. , Trebaljevac, J. , Obradovic, M. , Cenic‐Milosevic, D. , & Isenovic, E. R. (2017). Hormonal regulation of nitric oxide (NO) in cardio‐metabolic diseases. Current Pharmaceutical Design, 23, 1427–1434. 10.2174/1381612823666170124124855 28120715

[phy215300-bib-0028] Vigelsø, A. , Andersen, N. B. , & Dela, F. (2014). The relationship between skeletal muscle mitochondrial citrate synthase activity and whole body oxygen uptake adaptations in response to exercise training. International Journal of Physiology, Pathophysiology and Pharmacology, 6, 84–101. 25057335 PMC4106645

[phy215300-bib-0029] Wang, R. , Tian, H. , Guo, D. , Tian, Q. , Yao, T. , & Kong, X. (2020). Impacts of exercise intervention on various diseases in rats. Journal of Sport and Health Science, 9, 211–227. 10.1016/j.jshs.2019.09.008 32444146 PMC7242221

[phy215300-bib-0030] Widera, C. , Giannitsis, E. , Kempf, T. , Korf‐Klingebiel, M. , Fiedler, B. , Sharma, S. , Katus, H. A. , Asaumi, Y. , Shimano, M. , Walsh, K. , & Wollert, K. C. (2012). Identification of follistatin‐like 1 by expression cloning as an activator of the growth differentiation factor 15 gene and a prognostic biomarker in acute coronary syndrome. Clinical Chemistry, 58, 1233–1241. 10.1373/clinchem.2012.182816 22675198 PMC3539794

[phy215300-bib-0031] Wu, Y. , Zhou, S. , & Smas, C. M. (2010). Downregulated expression of the secreted glycoprotein follistatin‐like 1 (Fstl1) is a robust hallmark of preadipocyte to adipocyte conversion. Mechanisms of Development, 127, 183–202. 10.1016/j.mod.2009.12.003 20043993 PMC2849861

[phy215300-bib-0032] Xi, Y. , Gong, D. W. , & Tian, Z. (2016). FSTL1 as a potential mediator of exercise‐induced cardioprotection in post‐myocardial infarction rats. Scientific Reports, 6, 32424. 10.1038/srep32424 27561749 PMC5000295

[phy215300-bib-0033] Xu, X. , Zhang, T. , Mokou, M. , Li, L. , Li, P. , Song, J. , Liu, H. , Zhu, Z. , Liu, D. , Yang, M. , & Yang, G. (2020). Follistatin‐like 1 as a novel adipomyokine related to insulin resistance and physical activity. The Journal of Clinical Endocrinology and Metabolism, 105, dgaa629. 10.1210/clinem/dgaa629 32894773

[phy215300-bib-0034] Zheng, X. , Qi, C. , Zhang, S. , Fang, Y. , & Ning, W. (2017). TGF‐β1 induces Fstl1 via the Smad3‐c‐Jun pathway in lung fibroblasts. American Journal of Physiology‐Lung Cellular and Molecular Physiology, 313, L240–L251. 10.1152/ajplung.00523.2016 28495857

[phy215300-bib-0035] Zong, G. , Zhang, Z. , Yang, Q. , Wu, H. , Hu, F. B. , & Sun, Q. (2016). Total and regional apdiposity measured by dual‐energy X‐ray absorptiometry and mortality in NHANES 1999–2006. Obesity (Silver Spring), 24, 2414–2421. 10.1002/oby.21659 27667735 PMC5117479

